# The receptor for advanced glycation end products and risk of peripheral arterial disease, amputation or death in type 2 diabetes: a population-based cohort study

**DOI:** 10.1186/s12933-015-0257-5

**Published:** 2015-07-28

**Authors:** Jonas Malmstedt, Lars Kärvestedt, Jesper Swedenborg, Kerstin Brismar

**Affiliations:** Department of Molecular Medicine and Surgery, Karolinska Institutet, Stockholm, Sweden; Karolinska University Hospital, Stockholm, Sweden; Division of Vascular Surgery, Department of Surgery, South Hospital, 118 83 Stockholm, Sweden

**Keywords:** Peripheral arterial disease, Diabetes mellitus, Arteriosclerosis, Population studies

## Abstract

**Background:**

Patients with type 2 diabetes have a high risk for early and extensive development of peripheral arterial disease (PAD) and this excess risk is not explained by increased burden of traditional atherosclerotic risk factors. Activation of the receptor for advanced glycation end products (RAGE) could be one additional mechanism for accelerated PAD and increased risk for amputation and death. We investigated the association between RAGE plasma components and the risk for PAD, amputation and death in patients with type 2 diabetes. We also estimated the rate of amputation-free survival and survival without PAD.

**Methods:**

We investigated if plasma levels of carboxymethyl-lysine, S100A12 and endosecretory RAGE (esRAGE) were associated with two endpoints: survival without development of PAD and survival without amputation in a 12 years prospective population-based cohort of 146 patients with type 2 diabetes, free from PAD at inclusion. Influence of baseline plasma levels of RAGE ligands (individually and combined by a RAGE-score) were evaluated for both endpoints in the Cox-regression analysis.

**Results:**

106 patients survived without amputation and 93 survived without signs of PAD during follow up. Higher levels of S100A12 and RAGE-score were associated with increased risk for amputation or death, hazard ratios (HR) 1.29; 95% confidence interval (CI) [1.04, 1.59] and 1.79; 95% CI [1.07, 2.99] and with increased risk for PAD or death, HR 1.22; 95% CI [1.00, 1.49] and 1.56; [1.00, 2.44] after adjustment for age and sex. The effect was decreased after adjustment for Framingham cardiovascular disease score: risk for amputation or death, HR 1.17; 95% CI [0.94, 1.46] and 1.54; [0.95, 2.49], and risk for PAD or death, HR 1.12; 95% CI [0.91, 1.38] and 1.38; [0.91, 2.11] for S100A12 and RAGE-score respectively. The incidence for amputation or death was 2.8 per 100 person-years; 95% CI [2.0, 3.7] and the incidence rate for PAD or death was 3.6 per 100 person-years; 95% CI [2.7, 4.8].

**Conclusion:**

Higher plasma levels of S100A12 and the combined effect (RAGE-score) of esRAGE, carboxymethyl-lysine and S100A12 seem to be associated with shorter PAD- and amputation-free survival in patients with type 2 diabetes. This may indicate a role for S100A12 in PAD by activation of the RAGE system.

**Electronic supplementary material:**

The online version of this article (doi:10.1186/s12933-015-0257-5) contains supplementary material, which is available to authorized users.

## Background

Peripheral arterial disease (PAD) is a common macrovascular complication in patients with type 2 diabetes. Patients with PAD and diabetic complications, such as neuropathy, compromised infectious defence and microcirculatory changes, have a substantially increased risk for disability, amputation and death [1]. Furthermore, patients with diabetes are twice as likely to develop PAD, as compared to patients without, and progression from prediabetes to diabetes doubles the incidence of PAD [2].

Hyperglycaemia, the hallmark of diabetes, is independently associated with an increased risk for development of PAD [3–6]. Indeed, each 1% increase in glycated haemoglobin (HbA1c) was associated with a 28% excess risk for incident PAD at the end of 18 years follow up in the UK Prospective Diabetes Study (UKPDS) [5]. Despite the strong association with hyperglycaemia, several intervention trials looking at intensive glucose control failed to show reduction of macrovascular disease in patients with diabetes [7]. Furthermore, the increased occurrence of the traditional risk factors for atherosclerosis (smoking, dyslipidaemia and hypertension) cannot explain why patients with diabetes develop PAD early in life [8]. Hence, the increased predisposition to develop PAD in diabetes is multifactorial and new modifiable risk factors are sought for. Inflammation is one key process promoting atherosclerosis [9] and driving diabetic cardiovascular disease [10].

The receptor for advanced glycation end products is involved in chronic inflammation, diabetes and vascular disease. This receptor is expressed in endothelial and smooth muscle cells [11, 12]. The major exogenous ligands to RAGE are advanced glycation end products (AGE), a heterogeneous group of stable compounds involved in most diabetic complications [13]. AGEs are formed non-enzymatically during hyperglycaemia [14] and can also be derived by intestinal uptake from AGE-rich food [15]. *N*ε-(carboxymethyl)lysine (CML) is the predominant AGE found in tissue proteins [14] and an important ligand to the RAGE receptor [16].

S100-proteins, a family of proinflammatory proteins, are other endogenous ligands to RAGE [17]. A member of this family (S100A12 or calgranulin) shows elevated serum-levels in diabetes and is associated with overall mortality [18, 19]. Activation of RAGE has multiple effects in vascular tissue: it augments inflammation by causing release of cytokines, it stimulates vascular smooth muscle cell migration and proliferation and causes endothelial dysfunction and oxidative stress [12, 20–22]. There is ample animal evidence that stimulation of the RAGE signalling pathway amplifies atherosclerosis progression and inhibition of RAGE signalling prevents this process [23].

Endosecretory RAGE (esRAGE) is thought to act as a decoy receptor for advanced glycation end products and is consequently a beneficial form of RAGE [24]. esRAGE lacks the transmembrane domain of RAGE, and is produced by alternative splicing of RAGE mRNA [25]. Accordingly diabetic patients with various vascular complications exhibit low levels of esRAGE [18, 26–28], but the role in PAD is less clear [29].

Observational studies provide evidence for an association of the AGE-RAGE system plasma levels with mortality, coronary heart disease [19, 30, 31], and hyperglycaemia [28]. These studies are most often cross-sectional in design and mainly focused on type 1 diabetes or patients with end stage renal disease. Moreover, few studies included PAD patients and outcomes related to PAD, and those two who did were focused on patients with end stage renal disease [32, 33].

### Objective

Our objective was to investigate the role of AGE-RAGE in the development of macrovascular complications in patients with type 2 diabetes. The primary aim was to investigate if baseline plasma components of the AGE-RAGE system are associated with the risk of PAD or death and the risk for amputation or death. The second aim was to estimate the incidence of PAD-free survival and amputation-free survival in patients with type 2 diabetes in Sweden.

### Hypothesis

We hypothesized that patients with high levels of CML or S100A12 are more prone to develop peripheral vascular complications, compared to those with low levels. We also tested the hypothesis that high plasma levels of esRAGE are protective against the effects of activation of the AGE-RAGE system.

## Methods

### Study design and patients baseline data

We recruited 156 patients with type 2 diabetes from three primary healthcare centres with defined catchment areas, between July 1998 and May 2001. The study was approved by the regional ethics committee and written informed consent was obtained. Details of recruitment and study design are described elsewhere [34, 35].

We excluded patients with type 1 diabetes or late autoimmune diabetes of the adult (LADA). Diagnosis of LADA required positive glutamic acid decarboxylase antibodies and type 1 diabetes was defined as continuous need for insulin therapy before the age of 36. Patients with signs of PAD (loss of foot pulses or previous major amputation or revascularisation) at inclusion were excluded from analysis (n = 6), as detailed in Additional file 1: Table S1.

A single reviewer (LK) recorded baseline characteristics by questionnaire and review of medical records. Baseline examination included physical examination, recording of weight, length, hip- and waist circumference, blood pressure measurements and blood sampling. Patients were then followed for at least 10 years for mortality, major amputations and development of PAD.

### Sample collection

We collected venous blood in ethylene-diamine-tetra-acetic acid (EDTA) Vacutainer^®^tubes, (BD Diagnostics, Franklin Lakes, USA), and then immediately centrifuged them at 1,750*g* for 15 min to obtain platelet poor plasma which was stored at −70°C until analysis.

Human circulating esRAGE in plasma was measured by enzyme-linked immunosorbent assay (ELISA) technique using B-Bridge™ esRAGE ELISA Kit (Daiichi Fine Chemicals, Takaoka, Japan). Details are presented by Koyama et al. [27].

The endogenous ligand for RAGE, S100A12 was measured in plasma with CircuLex™ S100A12/EN-RAGE ELISA kit (MBL International Corporation, Woburn, USA, #CY-8058). The specific AGE-derivate CML was measured in plasma with CircuLex™ CML/*N*ε-(carboxymethyl)-lysine ELISA Kit (MBL International Corporation, Woburn, USA, #CY-8066). We followed the manufacturer’s instructions in all analyses.

### Follow up and endpoints

The primary endpoint was time from inclusion in study to major amputation (proximal to the fore-foot) or death, i.e. amputation-free survival. Secondary endpoint was death or development of PAD defined as any of: loss of foot pulse, development of ankle-brachial index <0.9, lower limb revascularization or major amputation during follow up. Four patients were lost to follow up due to migration, yielding 146 patients at baseline.

### Statistics

Continuous variables are presented as mean and 95% confidence interval (CI). We calculated confidence limits for event rates and proportions according to Bryar’s [36] and Wilson’s methods [37], using a Microsoft Excel spreadsheet file [38]. Cumulative event rates and survival curves were derived by the Kaplan–Meier method, using inclusion date as time zero. We evaluated the influence of individual AGE-RAGE plasma components on primary and secondary outcome by calculating the crude hazard ratio using a Cox proportional hazards model and subsequently adjusted the hazard ratio for age, sex and Framingham 10-year cardiovascular (CV) disease risk score [39]. This score has been validated in patients with type 2 diabetes and gives the individual 10-year sex-specific risk estimate for a CV event dependent on age, smoking, hypertension, systolic blood pressure, diabetes, LDL-cholesterol and HDL-cholesterol. We calculated the Framingham score for each patient at baseline using the Cox survival equation from the original publication [39]. We were thus able to control for all the traditional risk factors by including the CV risk score in our models. The compound effect of the AGE-RAGE-system on primary and secondary outcomes was evaluated by including a RAGE-score in the Cox models. We constructed the RAGE-score by averaging Ζ-standardized values [40] of plasma CML, S100A12 and esRAGE; (RAGEscore = [Ζ-CML+Ζ-S100A12 − Ζ-esRAGE]/3). We used the RAGE-score to avoid fitting models with too many variables in relation to sample size and because a possible interaction between these plasma proteins is not yet defined. A similar score has been used by others [33]. Patient age, plasma measurements, Framingham and RAGE scores were entered as continuous variables and all other variables were entered as categorical variables in the Cox models. Patients who survived without reaching an endpoint event before the end of follow up were considered censored. Forest plots with logarithmic scale for presentation of hazard ratio (HR) and 95% CIs from the Cox models were constructed using Meta Data Viewer software [41].

The proportional hazard assumption was tested by including a time-dependent interaction term in the model (likelihood ratio test) for continuous variables and by inspection of log-minus-log plots for categorical covariates.

All hazard ratios were reported with 95% CI and all tests were two-tailed.

## Results

### Baseline characteristics

The baseline characteristics of all patients are shown in Table 1. The median follow up time was 12.5 years; 95% CI [11.9, 13.1], equalling 1,540 person-years. The mean duration of diabetes at baseline was 6.9 years; 95% CI [6.2, 8.0] and 27%; [21, 35%] had insulin treatment. The majority was well controlled with respect to HbA1c 56; 95% CI [54, 58] mmol/mol (IFCC). Two thirds (66%) had peripheral neuropathy, 36% had retinopathy and 8.5% nephropathy at baseline. Hypertension and body mass index above normal were common. Thirteen patients had undergone coronary artery bypass surgery and four had had a stroke prior to inclusion.

**Table 1:** Baseline characteristics of patients initially without PAD and of those who did and did not survive without PAD during 12 years follow up

	All without PAD at baseline		PAD, amputation or death during 12 years follow up		Alive without PAD or amputation during follow up	
	N or mean	Proportion in %; [95% CI for proportion] or [95% CI for mean]	N or mean	Proportion in %; [95% CI for proportion] or [95% CI for mean]	N or mean	Proportion in %; [95% CI for proportion] or [95% CI for mean]
n	146	100%	53	36.3%	93	63.7%
Age (years)	61.6	[60.5–62.8]	64.2	[62.4–66.0]	61.2	[59.6–61.7]
Sex (male)	90	62%; [54–69%]	36	68%; [55–79%]	54	58%; [48–68%]
Diabetes duration (years)	7.1	[6.2–8.0]	7.9	[6.1–9.6]	6.7	[5.6–7.7]
Body mass index	29.3	[28.5–30.1]	28.8	[27.3–30.3]	29.7	[28.7–30.6]
Hypertension^a^	95	65%; [57–72%]	33	62%; [49–74%]	62	67%; [57–75%]
Systolic blood pressure (mmHg)	148	[145–152]	150	[144–157]	147	[142–151]
Present smoker	43	30%; [23–38%]	22	42%; [30–56%]^b^	21	23%; [15–32%]
Framingham 10-year CV risk score	37%	[33–40%]	46%	[40–52%]	32%	[29–35%]^c^
Retinopathy	36	30%; [22–38%]	16	36%; [23–50%]	20	26%; [17–37%]
Nephropathy^d^	12	8%; [5–14%]	5	9%; [4–20%]	7	8%; [4–15%]
Peripheral neuropathy	92	63%; [55–70%]	35	66%; [53–77%]	57	61%; [51–71%]
HbA1c mmol/mol (IFCC)	56	[54–58]	58	[54–63]	55	[52–57]
Cholesterol (mmol/L)	4.96	[4.79–5.13]	4.84	[4.52–5.16]	4.99	[4.79–5.20]
LDL-cholesterol (mmol/L)	3.08	[2.95–3.23]	3.02	[2.76–3.28]	3.09	[2.92–3.26]
HDL-cholesterol (mmol/L)	1.22	[1.16–1.28]	1.13	[1.02–1.23]	1.27	[1.19–1.34]
Triglycerides (mmol/L)	1.72	[1.52–1.92]	1.85	[1.40–2.29]	1.67	[1.47–1.87]
Creatinine (µmol/mL)	78	[75–82]	81	[73–89]	76	[73–80]
esRAGE (ng/mL)	0.32	[0.28–0.36]	0.30	[0.27–0.33]	0.32	[0.27–0.38]
S100A12 (ng/mL)	56	[41–72]	78	[37–119]	44	[33–55]
CML (µg/mL)	2.05	[1.67–2.42]	2.05	[1.25–2.86]	2.03	[1.63–2.43]

Data are N, proportion in%; [95% CI for proportion] *or* mean; [95% CI for mean].

*IFCC* International Federation of Clinical Chemistry.

^a^Blood pressure >130/80 or medication for hypertension.

^b^P = 0.014 (Pearson χ-square, 6.02).

^c^P < 0.0001 (T-test).

^d^Albuminuria >300 mg/L or S-creatinine above 100 mmol/L in women and 110 mm/L in men.

The mean Framingham 10-year risk for CV disease was 37%; 95% CI [33, 40%].

### Events during follow up

At end of follow up, 104 (71%) patients were alive without amputation, 2 (1.4%) alive with amputation, 39 (27%) dead without amputation, and 1 (0.7%) dead after amputation. The corresponding incidence rate for amputation or death was 2.8 per 100 person-years; 95% CI [2.0, 3.7].

Ninety-three (64%) patients were alive without PAD, 13 (9%) alive with PAD, 10 (7%) dead with PAD, and 30 (21%) dead without PAD. The incidence rate for PAD or death was 3.6 per 100 person-years; 95% CI [2.7, 4.8]. The incidence for PAD (censoring for death) was 16 per 1,000 person-years; 95% CI [10, 24].

Patients who survived without PAD or amputation had shorter diabetes duration and were slightly younger, less smokers, had lower Framingham 10 year CV risk score, less microangiopathy, tendency to better metabolic control, lower triglycerides and higher HDL (Table 1).

### Risk for amputation or death in relation to the AGE-RAGE system

**Fig. 1:**
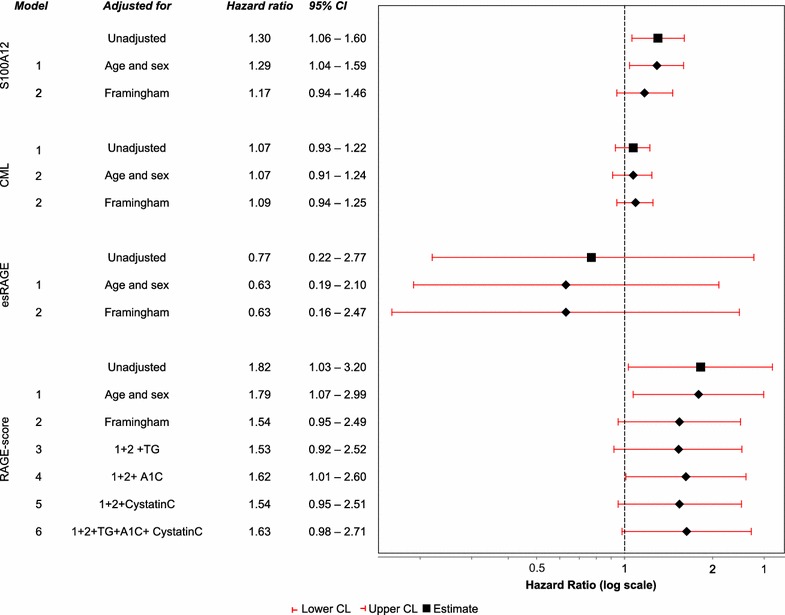
Estimated hazard ratios (Cox-regression) for major amputation or death in relation to RAGE system components. Hazard ratio (HR) >1.0 indicate increased risk for amputation or death (i.e. reduced amputation-free survival). HR for S100A12 is per 100 ng/mL increase and for RAGE-score per 1 unit (standard deviation). *Framingham* Framingham 10-years CV risk score, *TG* p-triglycerides.

Forty-two of 146 patients died or lost their leg during follow up. The risk for amputation or death in relation to RAGE components is illustrated in Fig. 1. S100A12 was the AGE-RAGE component with strongest association with amputation or death, HR 1.30; 95% CI [1.06, 1.60] per 100 ng/mL increase in S100A12. The increased risk for amputation or death was essentially unchanged after adjustment for age and sex. Further adjustment for Framingham CV risk score diminished the effect of S100A12, HR 1.17; 95% CI [0.94, 1.46]. The model with RAGE-score showed an increased risk, HR 1.82; 95% CI [1.03, 3.20] (unadjusted) and nearly similar risk when adjusted for age and sex, HR 1.79; 95% CI [1.07, 2.99]. Further adjustment for Framingham CV risk score diminished the effect of the RAGE-score, HR 1.54; 95% CI [0.95, 2.49]. The model with CML showed similar associations although weaker and with smaller effects, HR 1.07; 95% CI [0.93, 1.22] while esRAGE was not associated with amputation or death, HR 0.77; 95% CI [0.22, 2.77].

### Death and development of PAD in relation to the AGE-RAGE system

**Fig. 2:**
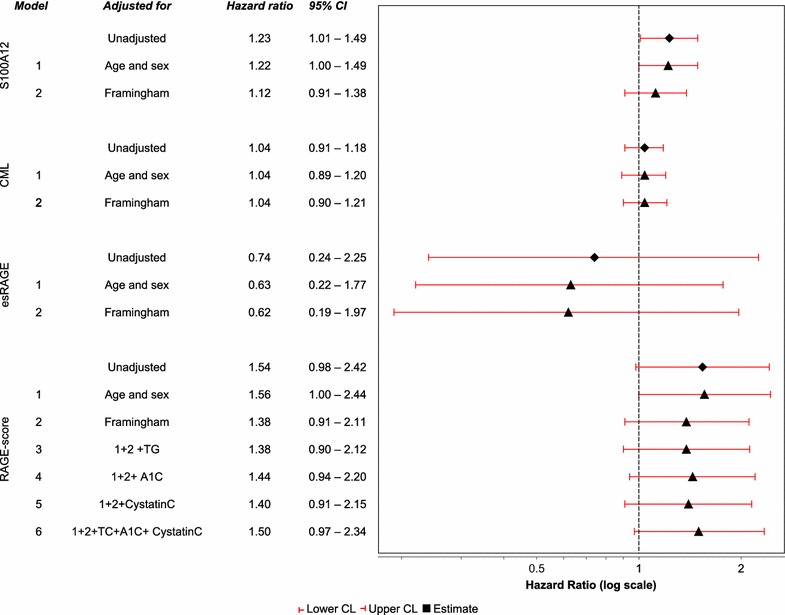
Estimated hazard ratios (Cox-regression) for development of PAD or death in relation to RAGE system components. Hazard ratio (HR) >1.0 indicate increased risk for developing PAD or death (i.e. reduced PAD-free survival). HR for S100A12 is per 100 ng/mL increase and for RAGE-score per 1 unit (standard deviation). *Framingham* Framingham 10-years CV risk score, *TG* p-triglycerides.

Fifty-three of 146 patients developed PAD or died during follow up. The risk for development of PAD or death in relation to RAGE components is illustrated in Fig. 2. Similar associations as were seen in amputation-free survival were seen in relation to PAD-free survival (Fig. 2). Higher plasma levels of S100A12 were associated with higher risk for development of PAD or death in both crude and age-sex adjusted models, HR 1.23; 95% CI [1.01, 1.49] and HR 1.22; 95% CI [1.00, 1.49] per 100 ng/mL increase in S100A12. Adjustment for Framingham CV risk score diminished the effect of S100A12, HR 1.12; 95% CI [0.91, 1.38]. The model with CML did not show any association with the outcome, HR 1.04; 95% CI [0.91, 1.18]. The model with RAGE-score showed an increased risk, HR 1.54; 95% CI [0.98, 2.42] (unadjusted), and nearly no change when adjusted for age and sex, HR 1.56; 95% CI [1.00, 2.44]. Further adjustment for Framingham CV risk score diminished the effect of the RAGE-score, HR 1.38; 95% CI [0.91, 2.11] (Fig. 2).

esRAGE was not associated with PAD-free survival neither in crude, HR 0.74; 95% CI [0.24, 2.25], or in age and sex adjusted models, HR 0.63; 95% CI [0.22, 1.77].

## Discussion

### Main findings: S100A12 and RAGE-score associated with outcome

We found plasma levels of S100A12 and RAGE-score being associated with amputation-free survival and development of PAD during a mean of 12-year follow up period in patients with type 2 diabetes. Higher levels of S100A12 and RAGE-score were associated with increased risk for amputation or death and higher S100A12 with development of PAD. These findings were not influenced by age or sex. Including Framingham 10-year CV risk score did attenuate the association. Our study is the first to investigate RAGE-score and S100A12 in relation to outcome in a population-based sample of patients with type 2 diabetes.

We used the RAGE-score to evaluate the combined effect of the RAGE-AGE system as the regulation and interactions between the individual components are not established. One previous study has shown S100A12 associated with mortality in patients receiving haemodialysis [42]. Our results are in line with previous evidence of elevated expression of S100A12 in coronary arteries from diabetic patients (compared to non-diabetic) [31], and that elevated plasma S100A12 has been associated with prevalence of cardiovascular disease [43] and PAD [32] in cross-sectional studies. Comparing plasma CML, S100A12 and esRAGE levels between different studies is almost impossible due to methodological problems such as whether levels of CML, esRAGE and S100A12 are measured in plasma or serum, methods used in analysis, and the lack of established reference levels. We consequently used platelet poor plasma, as this probably is the best estimate of physiological conditions, avoiding alterations caused by activated coagulation and platelet aggregation seen in serum samples.

### No influence of lipid levels

High triglycerides [44] have been shown to be a risk factor for CV disease especially in diabetes, but there is still some debate about to which extent and if there is a causative relationship or if triglycerides merely serve as a risk marker [45]. Including triglycerides in our regression models did not change the estimated effect of S100A12 and RAGE-score (data not shown).

HDL- and LDL-cholesterol are included in the Framingham risk score. Including Framingham score in our multivariate model may be erroneous as the AGE-RAGE system can be regarded as a very early event in the causal pathway to atherosclerosis [46], preceding some of the elements in the score such as elevated lipids and hypertension.

### CML and esRAGE showed no association with outcome

Plasma level of esRAGE is down-regulated in chronic hyperglycaemia; among RAGE ligands, the S100A12 protein, but not CML, appears to be associated with this effect [28].

We could not demonstrate any effect of esRAGE on outcome, probably due to low variation in esRAGE levels among our patients.

Higher levels of CML have been associated with incident fatal and nonfatal CV disease (including limb ischemia and amputations) as well as all-cause mortality in individuals with type 1 diabetes during long-term follow up [33]. The reason why we were unable to confirm this in our cohort with type 2 diabetes may be that our patients were younger with relatively short diabetes duration and a longer follow up may be needed. This is supported by the relatively low incidence of major amputations in our cohort.

### Mechanistic aspects

Thrombogenic abnormalities are involved in diabetic vascular complications including graft thrombosis [47].Citrated plasma could induce oxidative and inflammatory reactions in endothelial cells (EC) via the activation of thrombin-PAR-1 system and AGEs could potentiate the plasma-evoked EC damages via up-regulation of PAR-1 [48]. Blockage of the crosstalk between the thrombin-PAR-1 system and the AGE-RAGE axis by rivaroxaban may be a new therapeutic option to reduce thromboembolic events such as infrainguinal graft occlusion in patients with diabetes [48].

Moreover, AGE-RAGE-induced reactive oxygen species generation stimulates soluble dipeptidyl peptidase-4 release from EC, acting via the interaction with mannose 6-phosphate/insulin-like growth factor II receptor, and thus further potentiating the deleterious effects of AGEs in the vessel wall [49]. There is ample evidence from large studies on human atherosclerosis for a relationship between AGE levels and progression of coronary plaque burden, further emphasising an important role for AGE-RAGE axis in atherosclerosis [50].

Finally, genetic variants may be involved in regulation of the levels of esRAGE as genetic polymorphisms of fructosamine 3-kinase like rs1056534 and rs3848403 significantly correlate with sRAGE concentration in diabetic patients [51].

### Population-based incidence of PAD

There are scarce contemporary population-based data on the incidence rate for PAD in diabetic patients as most data come from cohorts derived from hospitals. Furthermore, criteria for PAD diagnosis, length of follow up and diabetes duration influence the estimates. Our estimate for incident PAD (16 per 1,000 person-years) is close to a similar study from Finland reporting new PAD in 21 of 107 patients followed for 11 years (i.e. ≈18 per 1,000 person-years) and the Atherosclerosis Risk in Communities (ARIC) Study with 14 per 1,000 person-years [52, 53]. The UK Prospective Diabetes Study (UKPDS) estimate for incident PAD during the first 6 years after diabetes diagnosis is 5 per 1,000 person-years. This lower incidence can be explained by younger age and shorter diabetes duration [5].

### Limitations

The limitation of this study is the low incidence of events and wide confidence intervals in the measured plasma components, making findings liable to be biased by confounding factors and measurement errors. We used pre-specified adjustment for possible confounders based on subject matter knowledge regarding causal factors for PAD instead of statistical driven covariate selection (i.e. “univariate screen”) [54]. Adjusting for Framingham score, which covers the traditional risk factors for CV disease, diminished the effect of the RAGE system as expected. One can argue that PAD can be defined in a more strict way, but neither ankle or toe pressures provide unequivocal diagnosis of PAD in type 2 diabetes.

### Strengths

One of the strengths of this study is a relatively unbiased patient population-based sample of people with diabetes instead of selected groups of patients (e.g. patients with end stage renal disease). Another strength is restriction to patients with type 2 diabetes and not Latent Autoimmune Diabetes of Adulthood (LADA) or type 1 diabetes. We believe that our results are largely valid and conclusions may be justified regarding the associations of S100A12 and RAGE components with amputation- and PAD-free survival, even if adjusted HR fall outside the commonly (miss)used significance limits (i.e. P < 0.05) [55–57]. We report point estimates with confidence intervals in order to give a more precise interpretation of the study results regarding both effect size and precision.

## Conclusion

The main importance of our results combined with aforementioned mechanistic and clinical studies is that the RAGE-AGE-system could play a role in development of PAD and for survival, but the associations seen may not represent causality.

Our findings suggest that the AGE-RAGE system plays a role in development of PAD in patients with type 2 diabetes. Higher S100A12 protein levels seems to be associated with decreased amputation-free survival but this finding needs to be explored in larger studies.

## Additional file

Additional file 1:
**Table S1.**Baseline characteristics for all patients in the original cohort. Data are means and SD (standard deviation) or n (%). *a* PAD defined as amputations or loss of foot pulse. *b* Albuminuria >300 mg/L or S-creatinine above 100 mmol/L in women and 110 mm/L in men. *c* Two patients had undergone both major and minor amputations. *d* P = .073 (Fisher’s Exact Test), *e* P = .072 (Fisher’s Exact Test), *f* P = .059 (Mann-Whitney U).

## Abbreviations

RAGE: receptor for advanced glycation end products
CML: *N*ε-(carboxymethyl)lysine
esRAGE: endosecretory RAGE
CI: confidence interval
HR: hazard ratio
SD: standard deviation
PAD: peripheral arterial disease
CV: cardiovascular
IFCC: International Federation of Clinical Chemistry
EDTA: ethylenediaminetetraacetic acid
ELISA: enzyme-linked immunosorbent assay
HbA1c: glycated haemoglobin
HDL: high-density lipoprotein cholesterol
LDL: low-density lipoprotein cholesterol
UKPDS: the UK Prospective Diabetes Study
