# Supplementary material for: The receptor for advanced glycation end products and risk of peripheral arterial disease, amputation or death in type 2 diabetes: a population-based cohort study
Source: Cardiovasc Diabetol. 2015 Jul 28;14:93. doi: 10.1186/s12933-015-0257-5 (PMC4517412; doi:10.1186/s12933-015-0257-5)
Supplement: Additional file 1: — Table S1.Baseline characteristics for all patients in the original cohort. Data are means and SD (standard deviation) or n (%). a PAD defined as amputations or loss of foot pulse. b Albuminuria >300 mg/L or S-creatinine above 100 mmol/L in women and 110 mm/L in men. c Two patients had undergone both major and minor amputations. d P = .073 (Fisher’s Exact Test), e P = .072 (Fisher’s Exact Test), f P = .059 (Mann-Whitney U). [file 12933_2015_257_MOESM1_ESM.docx]

# Supplementary table 1

#### Baseline characteristics for all patients in the original cohort

|  | All | With PAD *^a^* | Without PAD |
| --- | --- | --- | --- |
| *n* | 156 | 6 (3.80) | 150 (96.2) |
| *Demographic factors* |  |  |  |
| Age (years) | 61.7 (7.2) | 64.1 (4.1) | 61.6 (7.3) |
| Sex (male) | 95 (60.9) | 4 (66.7) | 91 (60.7) |
| Diabetes duration (years) | 7.2 (5.8) | 9.8 (9.9) | 7.1 (5.6) |
| Body mass index (kg/m^2^) | 29.2 (4.8) | 26.6 (2.0) | 29.3 (4.9) |
| *Risk factors* |  |  |  |
| Hypertension (>130/80 mmHg) | 102 (78.5) | 4 (66.7) | 98 (79.0) |
| Present smoker | 45 (29.2) | 2 (33.3) | 43 (29.1) |
| Cholesterol > 5 mmol/L | 81 (52.9) | 3 (50.0) | 78 (53.1) |
| Triglycerides > 1.7 mmol/L | 47 (30.7) | 4 (66.7) | 43 (29.3) |
| *Complications* |  |  |  |
| Retinopathy | 38 (28.8) | 2 (33.3) | 36 (28.6) |
| Nephropathy *^b^* | 13 (8.5) | 1 (16.7) | 12 (8.2) |
| Peripheral neuropathy | 98 (62.8) | 4 (66.7) | 94 (62.7) |
| Autonomic | 62 (39.7) | 2 (33.3) | 60 (40.0) |
| Motor | 31 (19.9) | 1 (16.7) | 30 (20.0) |
| Sensory | 48 (30.8) | 4 (66.7) *^d^* | 44 (29.3) |
| Amputations *^c^* | 2 (1.4) | 2 (33.3) | 0 |
| CABG | 14 (9.0) | 1 (16.7) | 13 (8.7) |
| Stroke | 4 (2.6) | 0 (0) | 4 (2.7) |
| *Laboratory data* |  |  |  |
| HbA_1C_ % | 6.4 (1.3) | 6.4 (1.3) | 6.4 (1.3) |
| LDL-cholesterol (mmol/L) | 3.06 (0.89) | 2.50 (1.36) | 3.09 (0.87) |
| HDL-cholesterol (mmol/L) | 1.22 (0.37) | 1.18 (0.41) | 1.22 (0.37) |
| Triglycerides (mmol/L) | 1.76 (1.24) | 2.74 (1.68) *^e^* | 1.72 (1.21) |
| *Research laboratory data* |  |  |  |
| esRAGE (ng/mL) | 0.32 (0.24) | 0.33 (0.19) *^f^* | 0.32 (0.24) |
| S100A12 (ng/mL) | 56 (96) | 58 (64) | 56 (97) |
| CML (µg/mL) | 2.03 (2.25) | 1.61 (0.71) | 2.04 (2.29) |

Data are means (SD) or n (%). *a* PAD defined as amputations or loss of foot pulse. *b* Albuminuria >300mg/L or S-creatinine above 100 mmol/L in women and 110 mm/L in men. *c* Two patients had had both major and minor amputations. d P=.073 (Fisher’s Exact Test), *e* P=.072 (Fisher’s Exact Test),
 *f* P=.059 (Mann-Whitney U).
